# Examining the adaptability and validity of interRAI acute care quality indicators in a surgical context

**DOI:** 10.1177/20503121221103221

**Published:** 2022-06-14

**Authors:** Timothy Wood, Mark Chatfield, Leonard Gray, Nancye Peel, Shannon Freeman, Melinda Martin-Khan

**Affiliations:** 1 School of Nursing, University of Northern British Columbia, Prince George, BC, Canada; 2 Centre for Health Services Research, University of Queensland, Brisbane, QLD, Australia

**Keywords:** Quality indicators, surgery, acute care, critical care, quality of care

## Abstract

**Background:**

Currently, the use of quality indicators in the surgical setting may be challenged by diverse patient needs, clinical complexity, and health trajectories. Therefore, the objective of this study was to examine the adaptability of existing quality indicators to a surgical context and propose new time points.

**Methods:**

A multi-method approach included an environmental scan of the literature, consultation with multinational experts, and analysis of surgical patient data. Quality indicators from the nurse-administered interRAI Acute Care instrument were examined within a surgical context using secondary data from a hospital in Brisbane, Australia (N = 1006 surgical cases).

**Results:**

A lack of relevancy of existing time points can preclude meaningful quality indicator measurement. Definitions of some quality indicators were adapted to ensure relevancy for the surgical population. As well, a surgical baseline (measured preoperative and post-injury) and a 48-h postoperative time point were added to the existing measurement timeline.

**Conclusion:**

Distinct measurement timelines were created for elective and non-elective surgical patients. The use of surgery-specific time points that can be embedded into an existing Acute Care measurement framework supports consistent quality indicator reporting. This study represents the first steps towards standardized quality reporting for health information systems across different care settings.

## Introduction

Surgical patients represent a heterogeneous group in the health care system who may be at increased risk to receive substandard care. The Institute of Medicine’s report, *To Err Is Human*,^1^ implicated surgery as a major factor in complex care where fragmentation in the health care system may increase patients’ risk of adverse events including as death. Surgical subgroups may be disproportionately affected. For example, the number of older adults who undergo surgical procedures continues to increase^2^ and it has been shown that older adults experience more postoperative complications compared to younger cohorts.^3^ Considering that some studies have identified surgical adverse events as accounting for the majority of all adverse events reported,^4,5^ it may be beneficial to focus quality improvement efforts on surgical patients as a means of enhancing patient safety and maintaining care consistency throughout the system of care.

Quality indicators (QIs) have been adopted and developed to examine the congruency between best practice and care delivery^6–10^ and commonly possess an explicit, agreed-upon definition that they be specific and sensitive to a given outcome; valid and reliable; relevant to the clinical question; permit comparison; and evidence-based.^11^ Organizations such as interRAI (www.interRAI.org) have developed instrument-specific QIs integrated within a standardized assessment including outcome measurement and quality improvement.^12,13^ The interRAI instruments, providing cross-service functionality through an integrated assessment system, retain the ability to identify and respond to opportunities to provide high-quality care.^14^

The interRAI Acute Care (AC) instrument is a nurse-administered assessment developed for older adults in AC settings.^12^ Reliability of interRAI AC items is well-recognized.^12,15,16^ In line with efforts to employ standardized assessment methodology to support an integrated health information system, it is necessary to consider contexts within the AC environment. Nine QIs are currently being used to measure quality of care in the interRAI AC instrument including (1) mobility, (2) pain, (3) bladder catheter, (4) cognitive health, (5) falls, (6) discharge to residential care, (7) prolonged length of stay, (8) skin integrity, and (9) self-care.^17^ These QIs are measured at three points in time: premorbid, admission, and discharge. Currently, the premorbid observation period is the 3 days preceding the acute illness leading to hospitalization. The admission time point is at admission to hospital prior to receiving any services, and discharge is measured at the conclusion of health care services immediately prior to exiting the hospital.

Given the varying levels of patient acuity, heterogeneity of health conditions and patient demographics, the role of emergency surgery, and varied time of onset of condition or trauma, the surgical patient population presents unique challenges to QI measurement. In the surgical context, particular health trajectories may not align with the time points currently in use for various AC QIs. Therefore, time points for QI measurement need to be examined with regards to the variable perioperative timeframe and associated volatility in both health state and functional status. In doing so, the adaptability of AC QIs for the nuances of surgical care may be better understood, laying the groundwork for the development of standardized QI measurement and comparison across services. Therefore, the objective of this study was to examine the adaptability of existing QIs to a surgical context and to propose new surgery-specific measurement time points to be included.

## Method

To examine the relevance and adaptability of interRAI AC QIs, a multi-method approach was used. This included three distinct phases: (1) environmental scan, (2) expert panel consultation, and (3) secondary data analysis of surgical cohorts.

### Phase 1: environmental scan

A brief environmental scan provided guidance on surgical timelines to be discussed with stakeholders. Three academic databases and additional grey literature were searched in 2018 using keywords and subject headings related to surgery, QIs, and surgical care trajectories. Databases included CINAHL, MEDLINE Ovid, and PsycINFO. Backwards searching of reference lists was performed to identify the relevant literature. Articles were initially screened by title and abstract for relevancy, after which articles underwent full-text review. Articles were included if they mentioned surgical QIs, if they were published in the past 10 years, and were in English language. Articles were excluded if they did not identify surgical timelines related to QI measurement or if they did not discuss how time points were developed and rationalized. Information gathered from the environmental scan was used to inform stakeholders of alternative measurement time points to be considered and propose new time points for use with the interRAI AC QIs.

### Phase 2: expert consultation

Semi-structured focus groups were conducted on seven separate occasions with key knowledge-holders which were purposively recruited from the Princess Alexandra Hospital Surgery Group in Brisbane, Australia (n = 5), and from the multinational group of interRAI researchers (n = 7). Potential participants were approached via email and through existing organizational networks. Inclusion criteria for participants were the following: have expert knowledge of interRAI or expertise in surgical QIs. Potential participants were excluded if they were unfamiliar with interRAI or had no knowledge of surgical QIs. Individuals with clinical expertise in surgical care (n = 3), geriatric medicine (n = 7), and physiotherapy (n = 2) participated. All individuals approached agreed to participate. Throughout the process of QI selection, reconceptualization of surgery-specific time points, and revision of applicable definitions, experts were regularly consulted by means of open-ended, non-structured engagement. Focus groups were conducted by two members of the research team (T.W., M.M.K.), both trained in qualitative research methods, in the workplace convenient to participants. Relationships were established with stakeholders prior to the focus groups by means of informal discourse and discussion of the study details. Two separate interviewers were used to minimize the risk of bias during consultation.

Five group consultations were face-to-face and two were via videoconference, typically lasting 30–60 min. Field notes were kept during focus groups by one of the researchers. To ensure comprehensiveness and accuracy of findings, consensus was obtained for selection of QIs requiring revision and subgroup-specific time points to be embedded. During face-to-face and videoconference consultations, information and prior work was presented to participants whereupon open discussion took place. Decisions were made via a group voting process, indicated by 100% agreement between stakeholders. In addition, ongoing correspondence between participants and the research team was encouraged outside of scheduled meetings to promote open communication.

A COREQ checklist has been included to report on qualitative findings (Supplementary material Table 2).

### Phase 3: statistical analysis

At the time of this study, nine outcome AC QIs could be calculated using data obtained from the interRAI AC^12^ and the interRAI Acute Care – Comprehensive Geriatric Assessment (AC-CGA)^17^ instruments. The interRAI AC QIs covers clinical, functional, and psychosocial domains and are used by decision-makers to support performance appraisal and comparison of the quality of services delivered.^18^ Using existing AC QIs and time points, descriptive analysis of two surgical cohorts was conducted. This included analysis of the Comprehensive electronic Geriatric Assessment (CeGA) clinical dataset (n = 814) and an AC research dataset (n = 192). Because of the limited surgical data containing information necessary for AC QI calculation, all cases designated as surgical in the datasets were deemed to have met inclusion criteria and were included in analysis; no restrictions were placed on examining specific surgical specialities. As such, no calculation for sample size was performed. Cases were excluded if they were not designated as surgical. The purpose of statistical analysis was to evaluate the applicability of AC QIs to two surgical settings, highlighting which QIs or time points may need to be revised. Proportions of health status indicators (e.g. mobility) were examined across multiple time points.

The University of Queensland confirmed ethics review was not required for this study (Exemption #2019000902). As well, because data are deidentified secondary data, informed consent was not necessary to obtain.

## Results

### Findings from the literature

A rapid review of the literature revealed that a paucity of research exists regarding time point development. After abstract and title screening, 346 articles were included for full-text review of which only three articles described the development process and rationale for measurement timelines of surgical QIs. Few studies in the extant literature discussed why particular time points were selected for QI measurement.^19–21^ Across included articles, a wide variety of surgical QIs exists. Furthermore, a lack of consistency in QI measurement challenges standardization. Drawing on the evidence that does exist, it was revealed that some time points may be applicable to existing QIs. In particular, it was identified that 48-h postoperative may represent an appropriate time point used for pain measurement,^21^ early bladder catheter removal, and assessment of mobility.^19,20^ While the use of the preoperative time period was often mentioned, specification of exactly when to collect data (e.g. pre-hospital admission, post-admission, or immediately preoperative) is limited.

### Findings from expert consultation

Several findings were generated from consultation with stakeholders, including the addition of surgery-specific time points, disaggregation of surgical patients into elective and non-elective, and the identification of QIs to be revised.

The original AC assessment tool has three time points (premorbid, admission, and discharge). Based on the literature reviewed and in collaboration with experts, two additional time points were recommended for surgical patients. These included an early-stay time point (preoperative that is post-injury and post-admission to hospital) and a mid-stay time point (48-h postoperative). It was found that these time points represent surgery-specific assessment periods that may yield useful data regarding the quality of care provided while accounting for health status volatility and short surgical timeframes. Despite the addition of early and mid-stay time points for surgical patients, discussion with focus groups revealed that two distinct subpopulations exist, each having unique pre-admission baseline statuses and post-surgical recovery trajectories which may further affect time point relevancy. These two cohorts are elective surgical patients and non-elective surgical patients. Reasons for the distinction included the relative medical stability and length of stay of elective patients (often electing for knee or hip surgery) versus the medical complexity or uncertainty in health trajectory that can be associated with non-elective surgery (e.g. traumatic injury requiring emergency surgery). Accordingly, surgical cohorts were divided into ‘elective’ and ‘non-elective’ with the goal of selecting only some or all of the time points for QI measurement based on the surgical subgroup (Table 1).

**Table 1.** Proposed surgical time points embedded into existing acute care quality indicator measurement structure.

	Time points	Surgical subgroup
TIME 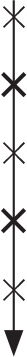	Premorbid – 3 days prior to onset of illness (leading to hospitalization)	(Non-elective)/(Elective)
	**Surgical Baseline – Preoperative (post-injury**)	(Non-elective)
	Admission – 24 h post admission	(Non-elective)/(Elective)
	**Admission T2 – 48 h postoperative**	(Non-elective)/(Elective)
	Discharge – discharged from acute care	(Non-elective)/(Elective)

Regular text = existing general medical time points. **Bolded and underlined text** = proposed surgery-specific time points.

QIs were divided into two groups: indicators that could be applied to the surgical setting without alteration, and indicators that required revision. Although the majority of AC QIs could be directly applied to the surgical setting (QIs: falls, cognitive health, discharged to residential care, skin integrity, prolonged length of stay), it was identified by experts that some required alteration to accommodate variation between surgical cohorts (QIs: bladder catheter, mobility, pain, self-care). Specifically, the premorbid (reflecting baseline health status) and admission periods were refined to reflect the distinctive health profiles and trajectories of surgical patients. Confirmation of the decision to adapt a QI was sought from the group of experts by means of consensus.

### Phase 3 findings: statistical analysis

The surgical sample from the CeGA dataset included 814 individual patient medical records with a mean age of 77 years (range: 32–102) and 46% male; admission assessments were only conducted once per patient. The mean length of stay in days was 44 (range: 2–280 days). Two-thirds of patients had not been hospitalized within the last 90 days prior to assessment, a relatively larger proportion, as compared with the other categories, who were in hospital (14%, n = 114). Similarly, two-thirds of patients reported time of onset of precipitating event within 0–7 days (65%, n = 530), whereas the next largest proportion was for 60 days or longer (19%, n = 154). For the AC dataset, there were a total of 192 surgical patients with a mean age of 79 years (range 70–96) and 40% male. The mean length of stay was 10 days (range 1–79). For this surgical cohort, a similarly large portion of patients had no hospitalization within the last 90 days (69%, n = 127). The time of onset of precipitating event was 0–7 days for 47% (n = 88) of the sample, whereas 35% (n = 66) had the health event more than 60 days prior to admission. For the AC dataset, surgical patients were able to be stratified by surgery type: low risk conservative (53%, n = 102), elective (27%, n = 52), and acute (20%, n = 38). A summary of sample characteristics of both datasets can be found in Supplementary material Table 1.

**Figure 1.**
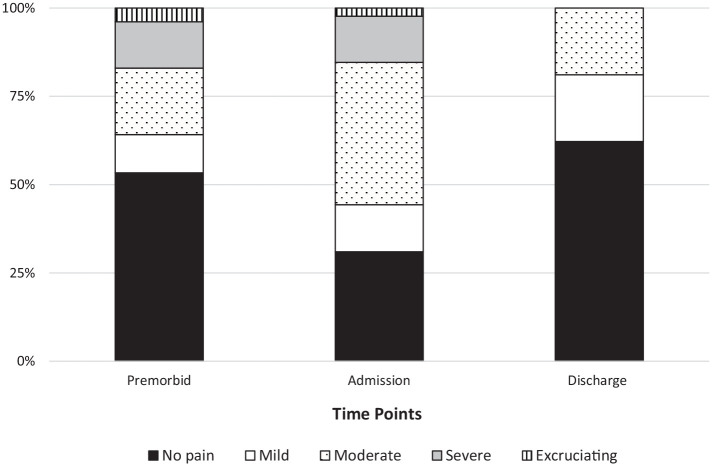
Comprehensive electronic geriatric assessment dataset–pain intensity by time point.

For CeGA patients, discharge data on health aspects of interest were collected on less than 5% of patients (n = 37). In addition, admission data were collected before or after actual admission time (mean 18 days post-admission). Across this dataset, the proportions of those with no pain changed substantially over the AC time points specified. There was a lower proportion of those with no pain at admission (31%) when compared with both discharge (62.2%) and premorbid (31%). For the moderate pain category, however, the reverse was observed: the proportion of those with moderate pain nearly doubled at admission (40.3%) as compared with both premorbid (18.8%) and discharge (18.9%) time periods (Figure 1).

**Figure 2.**
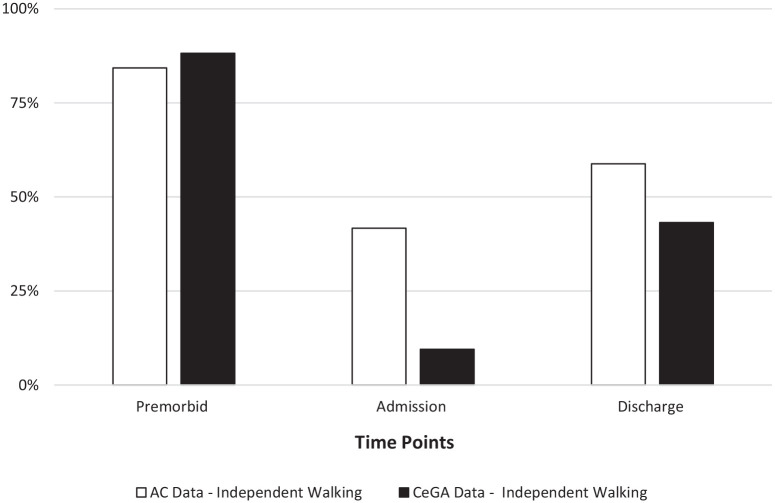
Independent Walking ADL by time point.

**Figure 3.**
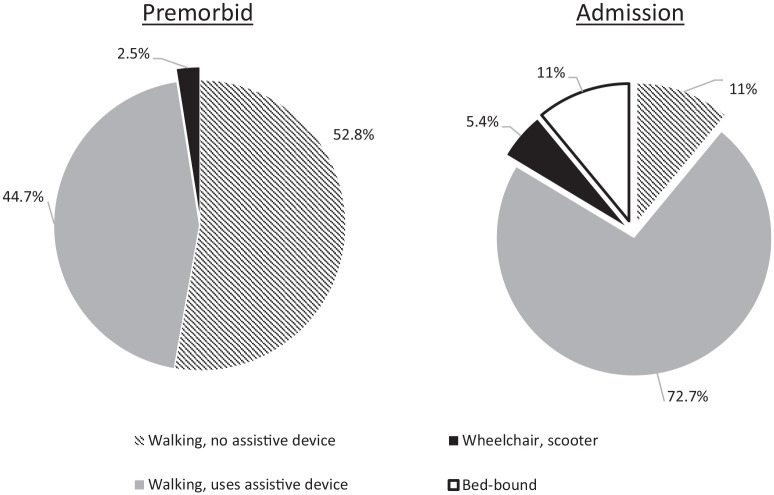
Comprehensive electronic geriatric assessment dataset – primary mode of locomotion by time point.

Similarly, the proportion of those who were classified as independent for the Walking ADL varied considerably across existing AC time points (Figure 2). Whereas 88.2% of individuals were walking independently premorbid, only 9.5% were independent at admission. At discharge, the proportion increased to 43.2%. Similar results were found for the AC dataset across the premorbid, admission, and discharge time points (84.3%, 41.7%, and 58.8%, respectively). See Table 2 for a complete breakdown of the Walking ADL categories across time points for both datasets. Examining the primary mode of locomotion in the CeGA dataset, the proportion of those walking with no assistive device changed substantially from 52.8% premorbid to 11% at admission (Figure 3).

**Table 2.** Walking ADL category by dataset.

Walking ADL category	AC data (n = 192)			CeGA data (n = 814)		
	Premorbid (n = 191)	Admission (n = 192)	Discharge (n = 187)	Premorbid (n = 812)	Admission (n = 812)	Discharge (n = 37)
Independent (%, (n))	84.3 (161)	41.7 (80)	58.8 (110)	88.2 (716)	9.5 (77)	43.2 (16)
Set-up help only (%, (n))	7.3 (14)	8.3 (16)	7.5 (14)	3.1 (25)	3.3 (27)	8.1 (3)
Supervision (%, (n))	3.7 (7)	9.4 (18)	5.7 (11)	4.2 (34)	22.9 (186)	32.4 (12)
Limited assistance (%, (n))	1.6 (3)	7.3 (14)	4.8 (9)	1.4 (11)	13.1 (106)	8.1 (3)
Extensive assistance (%, (n))	0.5 (1)	5.7 (11)	8.0 (15)	1 (8)	16.3 (132)	2.7 (1)
Maximal assistance (%, (n))	0 (0)	4.2 (8)	5.9 (11)	0.2 (2)	17.9 (145)	2.7 (1)
Total dependence (%, (n))	0 (0)	1.6 (3)	2.1 (4)	0.2 (2)	2.7 (22)	0 (0)
Activity did not occur (%, (n))	2.6 (5)	21.9 (42)	7.0 (13)	1.7 (14)	14.4 (117)	2.7 (1)

AC: Acute Care.

Through examination of these two surgical datasets in conjunction with stakeholder discussion, it was revealed that current AC time points may not adequately capture changes in surgical patient health status. The rapid changes in health status that occur in surgical cohorts (e.g. pain, mobility) require QI time points that can capture those changes.

## Discussion

Currently, existing AC QI time points are not sensitive to the nuances of the surgical context; the time between premorbid, admission, and discharge may be so long as to miss or ‘flatten out’ vital information for surgical patients, as demonstrated in secondary analysis of surgical cohorts. To adjust AC QIs to the surgical setting, it is necessary to account for the distinct trajectories of surgical subgroups and the relatively short timeframe for measurement and tracking of health status. By capitalizing on the utility of existing AC QI time points, this project emphasizes the value and need to adapt the interRAI AC QIs for the surgical setting and complement quality of care measures for surgical and general medical patients.

### Key considerations to inform the development of surgical QIs

#### Few QIs are unaffected by time points

Those QIs which focus on events in hospital and do not take into account the degree of decline or improvement over time are typically unaffected by the challenges of surgical timelines or differences in surgical trajectories. Examples include QIs such as ‘falls’ (the proportion of patients who fell at least once during the hospital episode) and ‘cognitive health’ (the proportion of patients with delirium-indicating behaviours at discharge) which are measured and reported at one point in time. Other QIs reporting on aspects of care that would not otherwise require revision for the surgical context include ‘newly discharged to residential care’, ‘prolonged length of stay’, and ‘skin integrity’.

#### Time points for distinct surgical trajectories

To adequately capture the nature of the surgical time point issue, individuals were disaggregated in a way that identifies the unique health trajectories associated with surgical care. While it may be reasonable to expect that most general medical patients who are admitted to the hospital should then be discharged with an equal (or better) level of mobility when compared with premorbid mobility status, this cannot be applied to some non-elective surgical patients. Considering that a number of orthopaedic trauma cases could necessarily result in a functional decline of some sort which is unrelated to the care provided in hospital, it is unreasonable to assume that these patients would return to their premorbid status by discharge. The full recovery process could take months or years, much of which occurs after surgical discharge.^22^ In addition, many patients are often appropriately discharged before they have reached ‘baseline’ status,^23,24^ which may artificially inflate the QI trigger rate. For example, full recovery to baseline ADL status for some elderly patients who undergo major surgery takes 6 weeks to 3 months.^25^ For these types of patients, existing AC QIs become less meaningful. Thus, the measurement timeline has to be revised to accurately reflect the anticipated effect of perioperative care on elective and non-elective surgical cohorts as two distinct groups.

Through extensive ongoing expert collaboration and consultation, surgery-specific time points were developed for both elective and non-elective subgroups. This included a surgical baseline measured preoperative (post-injury), and a 48-h postoperative time point. The preoperative surgical baseline was added as a means of introducing an ‘early-stay’ data time point to capture the immediate effect of injury (or health event) aside from the surgical care that follows. A 48-h postoperative time point was introduced as a ‘mid-stay’ data point to allow for the evaluation of the early effects of surgical treatment. A 48-h postoperative time point is supported by other studies examining mobility as a QI.^19,20^ The addition of these two time points sensitizes the QIs to the surgical context and allows for a distinction to be made between the effect of care while accounting for the effect of injury.

#### Utility of existing AC QIs

It has been recognized that the premorbid state is predictive of outcomes for a variety of health conditions.^26–28^ Thus, the role of the premorbid time point as it was initially conceptualized (3 days prior to illness) remains vitally important to provide a baseline measurement. The issue then becomes one of integration of new surgical time points into the existing measurement framework without redefining integral components. To isolate the effects of perioperative care, it is suggested that early and mid-stay time points be embedded as a means of sensitizing the existing measurement structure to the surgical setting. Including surgery-specific time points allows for the capturing of distinct surgical trajectories and supports use of standardized QIs across other AC settings.

#### Patient flow in the surgical setting

In the development of QIs for post-AC, Morris et al.^24^ note that full functional recovery cannot be expected in an acute hospital setting; patients are often discharged before the full healing process has taken place. This has important implications for the adaptation of AC QIs to surgery where data collection occurs at specified periods. Given the relatively short timeframe of perioperative care, the measurement timeline should coincide by utilizing corresponding time points to capture rapidly changing information. The postoperative period in particular is a time where there may be great volatility in health state with regard to a variety of quality measures (pain, mobility, self-care, bladder catheter). The substantial changes in health status as a result of both injury and surgical intervention directly affect QIs of interest. For example, examination of the walking ADL (as related to the mobility QI) revealed a substantial difference in the proportion of those walking independently between the premorbid and admission time points (Table 2). A similar trend was found by Wellens et al.^29^ who demonstrated fluctuations in ADLs from pre-admission to admission to discharge. These findings highlight the volatility of health status that can be associated with surgical patients, warranting the inclusion of early and mid-stay time points that coincide with surgical trajectories as opposed to simply revising QI definitions.

#### Surgical QI definitions

Based on the two time point additions, new definitions were also conceptualized for relevant QIs (Table 3). In line with other studies, we identified timely bladder catheter discontinuation as being important for the surgical context,^19,20^ as many surgical patients have catheters appropriately placed. The description of the bladder QI was adjusted as to reflect ‘timely removal’ as opposed to simply having a catheter newly placed (which would occur for many surgical patients). Pain was also altered according to the 48-h postoperative time point to capture the large changes in pain status that can happen over the perioperative period as demonstrated in our findings.

**Table 3.** Quality indicator definitions and proposed changes.

AC quality indicator	Variation	Existing definition	Proposed surgical definition by surgical subgroup
Cognitive health	Apply to all surgical patients; no definition change	The proportion of patients with delirium-indicating behaviours at discharge	*No change*
Falls		The proportion of patients who fell (at least once) during the hospital episode	*No change*
Discharge to residential care		The proportion of community-dwelling patients newly discharged to long-term care	*No change*
Prolonged length of stay		The proportion of patients with prolonged length of stay	*No change*
Skin integrity		The proportion of patients with a new or worsening pressure ulcer at discharge compared with admission	*No change*
Bladder catheter	Apply to all surgical patients; change definition	The proportion of female patients with a new urinary catheter on admission	**(Elective and Non-elective)** The proportion of female patients with a new urinary catheter present at 48 h
Pain	Apply to all surgical patients; change definition	The proportion of patients with no premorbid pain who reported both pain at admission and unimproved pain at discharge	**(Elective and Non-elective)** The proportion of patients with no premorbid pain who were discharged with unimproved pain when compared with reported postoperative pain at 48 h
Mobility	Create two cohorts (elective and non-elective); change definition for non-elective patients	The proportion of patients discharged with worse levels of walking compared with premorbid levels	**(Elective)** *No change* **(Non-elective)** The proportion of non-elective surgical patients with worse levels of walking between surgical baseline and discharge who also did not improve postoperative to discharge
Self-care	Create two cohorts (elective and non-elective); change definition for non-elective	The proportion of patients with pre-hospital decline who failed to return to pre-admission function (or better) by discharge	**(Elective)** *No change* **(Non-elective)** The proportion of non-elective surgical patients with a decline in function between surgical baseline and 48-h postoperative who fail to return to surgical baseline function (or better) by discharge

AC: Acute Care.

Sommer et al.^21^ identified a decrease in pain that occurs at 48-h postoperative until discharge; such changes would not otherwise be captured if using only the admission and discharge time points (as utilized by AC QIs). To align QI descriptions with the volatility associated with mobility status and other ADLs, both the 48-h postoperative and surgical baseline time points were used for mobility and self-care QIs. This is supported by McGory et al.’s^20^ suggestion of using both preoperative and 48 h postoperative measurement points, as well as Arora et al.’s^19^ use of the 48-h postoperative time point.

By combining revised definitions and the addition of surgically relevant time points according to patient-type, measurement timelines become sensitized to the surgical context. The use of embedded early and mid-stay surgical time points provides an opportunity to optimize consistency between quality measures to establish comparability between services and opens up new areas of inquiry into surgical care. This may enhance continuity of care and make progress towards a pan-service, standardized QI measurement system that is particularly relevant for those patients who move frequently between medical and surgical services.

### Next steps

To test the feasibility of surgical QI time points, it is necessary to conduct a pilot study including analysis of QIs on surgical cohorts from multiple sites using surgery-specific and existing AC QI time points. This may contribute towards the development of surgical QIs adapted from AC QIs which can facilitate consistent QI reporting across services.

### Limitations

Although analyses were descriptive in nature and datasets contained populations representing two different, yet similar, age groups, the purpose of the analysis was to examine proportions of health items over time for relevant QIs; the statistical analysis of AC QIs in surgical cohorts was supplementary to the main work. However, it must be noted that while data were inputted as admission-specific data, actual admission data were collected either before or after admission for the CeGA dataset. Alternatively, this strengthens conclusions about the volatility of health states in a surgical context particularly if the post-admission data are including the immediate postoperative period. In future studies, a more robust statistical analysis may help build on the foundational work presented here.

## Conclusion

Currently, existing AC QI time points are not usable in a surgical context given the rapid changes in health status that can occur. Given the heterogeneity of surgical patients, variable health trajectories, and existing utility of AC QIs, this project examines how surgical time points may be conceptualized and embedded into existing structures. By integrating measurement time points relevant to the surgical context, valuable information about changes in health status may be captured. The proposed time points and definitions were designed with the goal of pan-service consistency in QI measurement and have the ability to capture the granularity of the perioperative period. This project lays the groundwork for the development of a standardized QI measurement system that can enhance the continuity of care for all adult patients.

## Supplemental Material

sj-docx-1-smo-10.1177_20503121221103221 – Supplemental material for Examining the adaptability and validity of interRAI acute care quality indicators in a surgical context Click here for additional data file. Supplemental material, sj-docx-1-smo-10.1177_20503121221103221 for Examining the adaptability and validity of interRAI acute care quality indicators in a surgical context by Timothy Wood, Mark Chatfield, Leonard Gray, Nancye Peel, Shannon Freeman and Melinda Martin-Khan in SAGE Open Medicine
