# Supplementary material for: Examining the adaptability and validity of interRAI acute care quality indicators in a surgical context
Source: SAGE Open Med. 2022 Jun 14;10:20503121221103221. doi: 10.1177/20503121221103221 (PMC9201359; doi:10.1177/20503121221103221)
Supplement: sj-docx-1-smo-10.1177_20503121221103221 – Supplemental material for Examining the adaptability and validity of interRAI acute care quality indicators in a surgical context [file sj-docx-1-smo-10.1177_20503121221103221.docx]

**Supplementary Material**

**Table 1:** Sample Characteristics of Datasets

| **Variables** | **CeGA Data**  **(N=814)** | **AC Research Data**  **(N=192)** |
| --- | --- | --- |
| Age in years (mean [range]) | 77 (32-102) | 79 (70-96) |
| Male (%) | 46 | 40 |
| Length of stay in days (mean [range]) | 44 (2-280) | 10 (1-79) |
| Time since last hospital stay (%, [n])             No hospitalization within 90 days             31-90 days ago             15-30 days ago             8-14 days ago             In the last 7 days             Now in hospital | 67 (546)  9 (74)  3 (23)  3 (24)  4 (33)  14 (114) | *[N=183]*  69 (127)  12 (22)  6 (10)  7 (12)  4 (8)  2 (4) |
| Time of onset of precipitating event to admission (%, [n])             0-7 days             8-14 days             15-30 days             31-60 days             60+ days | 65 (530)  5 (44)  5 (43)  5 (43)  19 (154) | *[N=188]*  47 (88)  6 (11)  7 (13)  5 (10)  35 (66) |
| Surgery Type (%, [n])                   Low Risk conservative                   Elective                   Acute |  | 53 (102)  27 (52)  20 (38) |
